# Biopesticide Activity from Drimanic Compounds to Control Tomato Pathogens

**DOI:** 10.3390/molecules23082053

**Published:** 2018-08-16

**Authors:** Iván Montenegro, Alejandro Madrid, Mauricio Cuellar, Michael Seeger, Juan Felipe Alfaro, Ximena Besoain, Juan Pablo Martínez, Ingrid Ramirez, Yusser Olguín, Miryam Valenzuela

**Affiliations:** 1 Escuela de Obstetricia y Puericultura, Facultad de Medicina, Universidad de Valparaíso, Angamos 655, Reñaca, Viña del Mar 2520000, Chile; 2 Departamento de Química, Facultad de Ciencias Naturales y Exactas, Universidad de Playa Ancha, Avenida Leopoldo Carvallo 270, Playa Ancha, Valparaíso 2340000, Chile; 3 Facultad de Farmacia, Universidad de Valparaíso, Avenida Gran Bretaña 1093, Valparaíso 2340000, Chile; mauricio.cuellar@uv.cl; 4 Centro de Investigación Farmacopea Chilena (CIFAR), Universidad de Valparaíso, Santa Marta 183, Playa Ancha, Valparaíso 2340000, Chile; 5 Laboratorio de Microbiología Molecular y Biotecnología Ambiental, Departamento de Química & Centro de Biotecnología “Dr. Daniel Alkalay Lowitt”, Universidad Técnica Federico Santa María, Avenida España 1680, Valparaíso 2340000, Chile; michael.seeger@usm.cl (M.S.); felipealfaro88@gmail.com (J.F.A.); 6 Escuela de Agronomía Pontificia Universidad Católica de Valparaíso, Quillota, SanFrancisco s/n La Palma, Quillota 2260000, Chile; ximena.besoain@pucv.cl; 7 Instituto de Investigaciones Agropecuarias INIA Centro Regional La Cruz, Chorrillos 86, La Cruz 2280000, Chile; jpmartinez@inia.cl; 8 Centro de Biotecnología “Dr. Daniel Alkalay Lowitt”, Universidad Técnica Federico Santa María, Avenida España 1680, Valparaíso 2340000, Chile; ingrid.ramirez@usm.cl; 9 Center for Integrative Medicine and Innovative Science (CIMIS), Facultad de Medicina, Universidad Andrés Bello, Santiago 8320000, Chile; yusser.olguin@unab.cl

**Keywords:** polygodial, isonordrimenone, drimenol, clavibacter michiganensis, Pseudomonas syringae pv. tomato, Fusarium oxysporum, Phytophthora

## Abstract

Tomato crops can be affected by several infectious diseases produced by bacteria, fungi, and oomycetes. Four phytopathogens are of special concern because of the major economic losses they generate worldwide in tomato production; *Clavibacter michiganensis* subsp. *michiganensis* and *Pseudomonas syringae* pv. *tomato*, causative agents behind two highly destructive diseases, bacterial canker and bacterial speck, respectively; fungus *Fusarium oxysporum* f. sp. *lycopersici* that causes Fusarium Wilt, which strongly affects tomato crops; and finally, *Phytophthora* spp., which affect both potato and tomato crops. Polygodial (**1**), drimenol (**2**), isonordrimenone (**3**), and nordrimenone (**4**) were studied against these four phytopathogenic microorganisms. Among them, compound **1**, obtained from *Drimys winteri* Forst, and synthetic compound **4** are shown here to have potent activity. Most promisingly, the results showed that compounds **1** and **4** affect *Clavibacter michiganensis* growth at minimal inhibitory concentrations (MIC) values of 16 and 32 µg/mL, respectively, and high antimycotic activity against *Fusarium oxysporum* and *Phytophthora* spp. with MIC of 64 µg/mL. The results of the present study suggest novel treatment alternatives with drimane compounds against bacterial and fungal plant pathogens.

## 1. Introduction

The tomato (*Solanum lycopersicum* L.) is the most widespread vegetable, with the largest worldwide market. Its demand grows continually, as well as its cultivation, production, and trade [1,2]. The production increase during the latest decade is mainly due to performance improvements and, to a lesser extent, to more cultivated surfaces [3,4]. Worldwide production had a cumulative growth of 76.7% during the 2006–2010 period, according to the Food and Agriculture Organization of the United Nations (FAO) [5], with global production at 170.8 million tons in 2017 [6]. Similarly, consumption has continuously grown about 2.5% during the last 15 years [5]. In 2007, global tomato production represented a market of approximately US$68,000 million in exports, and continued to increase during 2007 [4]. Furthermore, global tomato imports have also shown an upward tendency, totaling US$7059 and US$8416 million in 2007 and 2011, respectively [4]. Given the economic importance of this crop, disease control is crucial for the expansion of the national tomato industry. The main tomato pathogens are bacteria; fungi; and to a lesser extent, oomycetes, which cause significant monetary losses. Bacterial diseases are the biggest problem for the tomato industry, with two major diseases affecting the health and growth of plants: bacterial canker and bacterial speck [7]. Both diseases represent a serious problem in both commercial plantings and home gardens [8]. Bacterial canker is caused by the Gram-positive bacterium *Clavibacter michiganensis* subsp. *michiganensis* (*Cmm*). This infectious disease can spread rapidly and result in devastating losses. It is particularly difficult to manage, because there is currently no cure for it, and it is difficult to eradicate once introduced into a greenhouse, garden, or field. The bacterial speck of tomato is caused by *Pseudomonas syringae* pv. tomato (*Pst*), a Gram-negative bacterial pathogen. Like many syringae pathovars, *Pst* grows epiphytically on a wide range of plants, although field populations decline in the absence of a susceptible host. Although serious disease outbreaks are infrequent, conditions such as high leaf wetness, cool temperatures, and some cultural practices favor the dissemination of *Pst* between host plants [9]. The dissemination of the bacteria in plants causes a low development of the fruit, which produces a short post-harvest period and thus causes decreases in tomato exports. As another risk, fungal diseases can cause strong losses for tomato production. Some fungi have a broad host range and probably circulate between commercial crops and wild plants. This increases the likelihood of fungus spreading into new areas and represents a constant threat. Vascular wilting of the tomato caused by the fungus *Fusarium oxysporum* f. sp. *lycopersici* (*Fol*), a deuteromycete pathogen, is a worldwide phytosanitary problem limiting tomato production [10]. There is not currently a successful method of chemical control for this pathogen, although it can be moderately limited by a combination of good farming practices, resistant cultivars, soil solarization, and fumigation [11]. Infections caused by oomycetes are globally distributed and prosper in a wide range of environments. Pathogenic species that live in association with plants or animals can cause serious diseases and eventually destroy their hosts [12]. Oomycete infections caused by *Phytophthora* spp. (*Pp*) represent a major threat to natural forests and some crop plants. In particular, *Phytophthora infestans* is a foliar pathogen responsible for late blight, a disease that affects potato and tomato crops worldwide.

Drimanic compounds, which are sesquiterpenes with bicyclic farnesane-type skeletons. Several important bioactivities, such as antimicrobial [13], antifouling [14], cytotoxic [13,15], and insect antifeedant [16,17] activities. The drimanic compounds polygodial, drimenol, isonordrimenone, and nordrimenone possess antifungal activity [18,19,20]. Polygodial is the most widely occurring sesquiterpene dialdehyde, which have been reported worldwide in flowering plants, ferns, fungi, and marine molluscs [21]. Conformational studies of these compounds demonstrate that the drimanic skeleton and the Δ7,8-double bond are key structural features related to the antifungal activity [20]. Recently, it has been reported that polygodial affects growth of normal and resistant isolates of *Botrytis cinerea* with the concentration of a drug that gives half-maximal response (EC_50_) values ranging between 117 and 175 ppm.

The aim of this study is to evaluate the effects of drimanic compounds s against phytopathogens that strongly affect tomato cultivation.

## 2. Results and Discussion

**Figure 1:**
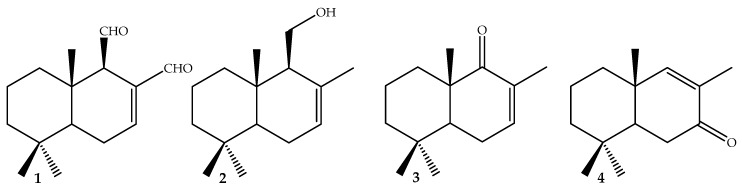
Chemical structures of drimanes: (**1**) polygodial, (**2**) drimenol, (**3**) nordrimenone, and (**4**) isonordrimenone.

Herein, we discuss the results obtained in a study of anti-phytopathogenic activity of compounds **1**–**4** against microorganisms *Cmm*, *Pst*, *Fol*, and *Pp*. Figure 1 shows these four drimane sesquiterpenes compounds, obtained from secondary metabolites and derivatives of *D. winteri*, which has previously been shown to have interesting biological activity.

The antibacterial activity (Table 1) was evaluated against *Cmm* and *Pst*, while antifungal activity (Table 2) was evaluated against *Fol* and *Pp* at different concentrations of compounds **1**–**4**.

Compound **1** showed higher activity against *Cmm*, comparable with copper sulfate pentahydrate (CuSO_4_·5H_2_O), with MIC and minimum bactericidal concentrations (MBC) values of 16 and 64 µg/mL, respectively. In addition, compound **1** was potently active against Gram-negative bacteria *Pst*, with MIC and MBC values of 32 µg/mL. Compound **4** showed activity against both *Pst* (MIC and MBC, 64–128 µg/mL) and *Cmm* (MIC and MBC, 32–64 µg/mL).

Anti-oomycete results revealed that polygodial (**1**) showed strong activity against *Pp*, comparable with triazolic antimycotic difenoconazole, with MIC and minimal fungicidal concentration (MFC) values of 64–128 µg/mL. However, compound **1** presented the same antimycotic effects against *Fol*, with MIC and MFC values of 64–128 µg/mL. The effectiveness of nordrimane compound **4** presented moderate activity against *Fol*, with MIC and MFC values of 128 µg/mL (see Table 2).

Polygodial (**1**) possessed completely disruptive effects against fungal cells, while **4** showed moderate effects. Minor effects were observed for compound **2**, and compound **3** showed no disruptive effects (See Table 3).

This study evaluated the anti-bacterial, anti-fungal, and anti-oomycete activity of four drimane compounds. Antimicrobial activity analysis of the drimane compounds showed the most pronounced anti-phytopathogenic activity in compounds **1** and **4**.

Most drimane and nordrimane α,β-unsaturated dialdehydes possess a wide variety of biological activities including antibacterial, antifungal, anti-inflammatory, anti-tumor, cytotoxic, and phytotoxic activities [13,15,22,23].

Anke reported the antibacterial activity of compound **1** against Gram-negative and Gram-positive bacteria at minimal inhibitory concentrations of 2–20 µg/mL [13]. Kubo also reported that compound **1** exhibited moderate antibacterial activity against both Gram-positive bacteria such as *Bacillus subtilis* and *Staphylococcus aureus*, with minimum bactericidal concentrations (MBC) values of 100 µg/mL and Gram-negative bacteria such as *Salmonella choleraesuis* and *Escherichia coli*, with MBC values of 50 µg/mL and 100 µg/mL, respectively [24]. 

This study obtained different MIC and MBC values for compound **1** against *Cmm* and *Pst* (Table 1). Because *Cmm* is a Gram-positive Actinobacteria and *Pst* is a Gram-negative bacteria, the difference in MIC and MBC values is likely due to lipid membrane contents, and may be associated with differences in the cell wall structure. The peptidoglycan is 90% of the cell wall in Gram-positive bacteria and only 10% of the cell wall in Gram-negative bacteria. Lipid matrices in plasma membranes contain a variety of lipids, among which phosphatidylethanolamine (PE) and phosphatidylserine (PS) contain a primary amine group [24]. Cimino, Fujita, and Kubo suggest that the mechanism of action for compound **1** may form a pyrrole with the amine groups in PE and PS on the plasma membrane outer leaflet, thereby disturbing the balance of the plasma membrane [24,25,26].

Next, compound **4** presented antibacterial effects, confirming the chemically high reactivity of the α,β-unsaturated ketone group system in Michael reactions. This compound was previously reported to have antifungal activity [19]. Compound **3** did not present antibacterial activity against *Cmm* below concentrations of 256 µg/mL. 

Montenegro reported on *D. winteri* extract effects against pathogenic yeasts that cause alterations in cell membranes [27]. Other studies suggest that certain compounds extracted from, for example, *Polygonum acuminatum* Kunth and *Pseudowintera colorata* possess antibacterial and antifungal properties [24,28], including compounds **1** and **2**. Compound **1** is an unsaturated sesquiterpene dialdehyde known to possess anti-fungal activities and to induce membrane damage [18,20,24,29]. The antifungal and antibacterial properties of **1** come from its ability to function as a non-ionic surfactant, disrupting the lipid–protein interface of integral proteins and denaturing their conformation in eukaryotic cells [23,24]. Kubo and Castelli propose that **1** inhibits plasma membrane H^+^-ATPase by disrupting and disorganizing hydrogen bonds at the lipid bilayer protein interface [18,30]. The same authors indicate that reactive oxygen species (ROS) production induced by **1** is similar to that of menadione, though much lower than that of compound **2**. A recent morphological study reported by Carrasco indicates that conidia treated with polygodial have smaller sizes and irregular membrane borders, confirming the different mechanism of action in the two compounds (although that study does not discuss how membrane disruption occurs) [28]. To continue, **2** is a drimane homoallylic alcohol that has been shown to have antifungal activity against *B. cinerea* [18,23,24,31,32]. Nordrimenone (**3**) is a drimane-type nor-sesquiterpene that has been shown to possess antifungal activity against *C. albicans, T. rubrum* CCC 110*, T. mentagrophytes* ATCC 9972, and *M. gypseum* CCC 115 [19]. Isonordrimenone (**4**) has been identified in tobacco plants as a nordrimane sesquiterpene, and was previously shown to present antifungal activity against dermatophyte fungi, pathogenic yeasts, and encapsulated yeast *C. neoformans* [20].

In short, the findings in this study are consistent with previous studies, showing that drimane unsaturated dialdehyde **1,** with a Δ7,8 double bond, and α, β-unsaturated nordrimane **4**, with a Δ8,9-double bond, induce antimicrobial effects, damage fungal cells, and affect oomycete membranes.

## 3. Materials and Methods 

### 3.1. General

Solvents, synthesis reagents, biological reagents, and positive controls were obtained from Aldrich (St. Louis, MO, USA) and were used without further purification. A detailed description of conditions used to register Fourier transform infrared (FT-IR) spectra, high resolution mass spectra, and ^1^H, ^13^C, ^13^C DEPT-135, gs-2D heteronuclear single quantum coherence (HSQC) and gs-2D HMBC spectra have been given [20]. Column chromatography (CC) was performed with silica gel 60 from Merck (Darmstadt, Germany), and thin layer chromatography (TLC) was carried out on precoated silica plates F-254 from Merck (Darmstadt, Germany). Melting points were determined on an electrothermal instrument and are presented uncorrected. 

### 3.2. Isolation, Synthesis, and Characterization of Compounds ***1***–***4***

Polygodial (**1**) and drimenol (**2**) were isolated from dichloromethane extract of D. winteri bark. The extraction methodology and isolation of pure compounds were performed according to reported procedures [28]. Isonordrimenone (**3**) and nordrimenone (**4**) were synthesized according to procedure reported by Montenegro [22]. Compounds **1**–**4** (Figure 1) were identified by melting point, optical rotation, and spectroscopic data, including ^1^H- and ^13^C-NMR and comparisons with data reported in the literature [33,34].

### 3.3. Microorganisms

The microbial strains of *Pst*, *Cmm* [35], *Pp*, and *Fol* were received from the Laboratory of Phytopathogy, Escuela de Agronomía, Pontificia Universidad Católica de Valparaíso.

### 3.4. In Vitro Antibacterial Assay

The antibacterial activity of the compounds **1**–**4** was evaluated using broth macrodilution methods [36,37] in two-fold; serial dilutions in 1 mL of King´s B medium (KB) for *Pst*, and yeast–peptone–glucose broth (YPGB) for *Cmm*. Antimicrobial substances were dissolved in DMSO (5 mg/mL), and added to KB or YPGB at concentrations of 1, 2, 4, 8, 16, 32, 64, 128, and 256 µg/mL. Sterile water and DMSO were used as negative controls. Copper sulfate pentahydrate (CuSO_4_·5H_2_O) was used as positive control. The bacterial inoculums were prepared from an “overnight” culture, adjusting the concentration to 1–5 × 10^6^ CFU/mL. Ten microlitres from every dilution will be surface inoculated on KB agar or YPGA, respectively, at 0, 24, 48, and 72 h (two replicates). The minimal inhibitory concentrations (MIC) and minimum bactericidal concentrations (MBC) were defined as the lowest concentrations of compound at which microbial growth was inhibited (MIC), or reduced by 99.9% (MBC), respectively, after 72 h incubation in a rotatory shaker at 28 °C.

### 3.5. Effect of Compounds on Mycelia Growth of Phytophthora and Fusarium

*Pp* and *Fol* cells were grown on PDA in 9 cm Petri plates for 14 days at 20 °C and 25 °C, respectively. Mycelial plugs (7 mm diameter) excised from the edge of an actively growing colony was transferred to the center of each 6 cm Petri plate containing PDA amended with the molecules at following concentrations: 200, 150, 125, 100, 75, 50, 25, 12.5, 6.25, and 3.125 and 0 μg/mL. Antimicrobial substances were added to the PDA medium after autoclaving when the medium had cooled to 55 °C. Plates containing PDA without antimicrobial substances were included as negative controls. For each substance and concentration, three replicate plates were used. The plates were incubated in darkness at 20 °C for *Pp* and 25 °C for *Fol*. Colony diameter was measured after seven days in two perpendicular directions on each plate. The diameter of the mycelial plug inoculum was subtracted and the two diameter measurements were averaged. Percent growth inhibition for each antimicrobial substance and concentration was calculated by dividing colony diameter in the treated plates by that in the control plates (no antimicrobial substance added) [38].

### 3.6. Measurement of Cellular Leakage 

Fungi cell leakage was assessed according to Lunde and Flores by measuring 260-nm-absorbing materials released to the medium, primarily representing nucleotides of which uracil-containing compounds exhibited the strongest absorbance [39,40]. *Pp* and *Fol* cells cultured by shaking at 30 °C to the early stationary phase were washed two times and diluted to approximately 5 × 10^7^ CFU/mL with cold MOPS buffer, pH 6.0. Cells were aliquoted to tubes, and 100 µg/mL of compounds **1**–**4** and difenoconazole were added. Instead, sodium dodecyl sulfate (SDS) was utilized at a final concentration of 2% to produce 100% cell lysis. Then, cells were incubated stationary at 30 °C, samples were taken at intervals and spun at 8000× *g* for 5 min in microcentrifuge tubes, and the supernatants were collected for analysis. The results were presented as the means of values from at least two independent assays.

### 3.7. Statistical Analysis 

Statistical analysis of recovery rates was performed by comparison within isolates and between culturing media used with a Student’s *t*-test. Differences were considered significant at *p* < 0.05. 

## 4. Conclusions

This study evaluated anti-phytopathogenic activity of four drimane compounds against *Cmm, Pst*, *Fol*, and *Pp*. The results show that compound **1** affects mycelial growth and powerfully inhibits germination of *Pp* and *Fol*. The mechanism of action of compound **1** involves membrane damage. Comparatively, compound **1** is more effective against bacterial and fungal phytopathogens than compound **4**. Regardless, the results show that research on drimane compounds can generate new applications with important impacts for productive sectors, and specifically the tomato crop industry.

## Figures and Tables

**Table 1:** Minimal inhibitory concentrations (MIC) and minimum bactericidal concentrations (MBC) values ^a^ of compounds **1**–**4** against bacterial strains.

Compounds	MIC (µg/mL)		MBC (µg/mL)	
	*Cmm*	*Pst*	*Cmm*	*Pst*
**1**	16	32	64	32
**2**	256	>256	>256	>256
**3**	256	>256	>256	>256
**4**	32	64	64	128
**CuSO_4_·5H_2_O ^b^**	25	12.5	100	25
**Dimethyl sulfoxide (DMSO ^c^)**	i	i	i	i

^a^ Each value represents the mean ± standard deviation (SD) of three experiments, performed in triplicate. ^b^ Positive control. ^c^ Negative control. i. Inactive compound.

**Table 2:** The MIC and MFC values ^a^ of the compounds **1**–**4** against oomycete *Pp* and fungus *Fol*.

Compounds	MIC (µg/mL)		MFC (µg/mL)	
	*Pp*	*Fol*	*Pp*	*Fol*
**1**	64	64	128	128
**2**	>256	128	>256	256
**3**	>256	256	>256	>256
**4**	128	128	128	128
**Difenoconazole ^b^**	100	100	150	125
**Metalaxyl ^b^**	10	125	20	150
**DMSO ^c^**	i	i	i	i

^a^ Each value represents the mean ± SD of three experiments, performed in triplicate. ^b^ Positive control. ^c^ Negative control. i. Inactive compound.

**Table 3:** Cellular leakage effects of compounds **1**–**4** against oomycete *Pp* and fungus *Fol*.

Compounds	%Damage ^a^	
(100 µg/mL)	*Pp*	*Fol*
**1**	100	100
**2**	0	30
**3**	0	0
**4**	70	80
**Difenoconazole ^b^**	100	100
**SDS ^c^**	100	100

^a^ Damage produced by compounds **1**–**4** compared with damage produced by sodium dodecyl sulfate (SDS). ^b^ Positive control. ^c^ SDS ionic detergent (chaotropic agent) was utilized at a final concentration of 2% to produce 100% cell lysis.
